# Unplanned return presentations of older patients to the emergency department: a root cause analysis

**DOI:** 10.1186/s12877-020-01770-x

**Published:** 2020-09-22

**Authors:** Babiche E. J. M. Driesen, Hanneke Merten, Cordula Wagner, H. Jaap Bonjer, Prabath W. B. Nanayakkara

**Affiliations:** 1 Department of Emergency Medicine, Amsterdam UMC, Vrije Universiteit Amsterdam, De Boelelaan 1117, Amsterdam, 1081 HV The Netherlands; 2 Department of Public and Occupational Health, Amsterdam Public Health Research Institute, Amsterdam UMC, Vrije Universiteit Amsterdam, Van der Boechorststraat 7, Amsterdam, 1081 BT The Netherlands; 3 Netherlands Institute for Health Services Research (NIVEL), Otterstraat 118-124, Utrecht, 3513 CR The Netherlands; 4 Department of Surgery, Amsterdam UMC, Vrije Universiteit Amsterdam, De Boelelaan 1117, Amsterdam, 1081 HV The Netherlands; 5 Department of Internal Medicine, Amsterdam Public Health Research Institute, Amsterdam UMC, Vrije Universiteit Amsterdam, De Boelelaan 1117, Amsterdam, 1081 HV The Netherlands

**Keywords:** Acute care, Emergency department, Older patients, Elderly, Preventability, Return presentations, Root causes

## Abstract

**Background:**

In line with demographic changes, there is an increase in ED presentations and unplanned return presentations by older patients (≥70 years). It is important to know why these patients return to the ED shortly after their initial presentation. Therefore, the aim of this study was to provide insight into the root causes and potential preventability of unplanned return presentations (URP) to the ED within 30 days for older patients.

**Methods:**

A prospective observational study was conducted from February 2018 to November 2018 in an academic hospital in Amsterdam. We included 83 patients, aged 70 years and older, with an URP to the ED within 30 days of the initial ED presentation. Patients, GPs and doctors at the ED were interviewed by trained interviewers and basic administrative data were collected in order to conduct a root cause analysis using the PRISMA-method.

**Results:**

One hundred fifty-one root causes were identified and almost half (49%) of them were disease-related. Fifty-two percent of the patients returned to the ED within 7 days after the initial presentation. In 77% of the patients the URP was related to the initial presentation. Patients judged 17% of the URPs as potentially preventable, while doctors at the ED judged 25% and GPs 23% of the URPs as potentially preventable. In none of the cases, there was an overall agreement from all three perspectives on the judgement that an URP was potentially preventable.

**Conclusion:**

Disease-related factors were most often identified for an URP and half of the patients returned to the ED within 7 days. The majority of the URPs was judged as not preventable. However, an URP should trigger healthcare workers to focus on the patient’s process of care and their needs and to anticipate on potential progression of disease. Future research should assess whether this may prevent that patients have to return to the ED.

## Background

There is a steady increase in the number of older patients (age ≥ 70 years) presenting at the Emergency Department (ED) each year, accounting for up to 30% of all ED presentations worldwide [1]. This growing group of older patients is mentioned as one of the reasons why ED crowding occurs. ED crowding is a major challenge worldwide, and has negative consequences on the efficiency, quality and safety of emergency care.

After an ED presentation, more than one in four patients returns to the ED at least once during the following year [2]. This group of patients accounts for almost half of all the ED presentations [2]. These return presentations have negative consequences for both the patient and healthcare system (i.e. higher healthcare costs).

Previous literature shows that return presentations to the ED within 30 days occur in 20% of the older patients [3] and at 6 months return presentation rates are as high as 40% [4]. Older patients often present with atypical medical and psychosocial issues and have multiple comorbidities [5, 6]. Griffin et al. [7] concluded that frequent ED users have a higher mortality and that age and comorbidity are significant predictors of mortality. The average 30-day mortality rate of older patients following an ED presentation is 10% [1] indicating the potential fragile state they are in [6]. In order to reduce unplanned return presentations (URPs) of older patients to the ED, greater understanding is needed on what factors influence the utilization of the ED and what interventions could have potentially prevented URPs. So far, little is known about the causes of the return presentations. In the literature, preventability is measured in different ways; based on combinations of diagnostic codes between the initial and return presentation, judged by independent reviewers, or seen through a patient or doctor perspective [8–12]. In our opinion, the underlying causes and potential preventability of an URP to the ED are not limited to the ED and therefore the preventability should be judged from different perspectives involving healthcare workers from the acute care chain. Therefore, this study aims to provide insight from a broader perspective, asking the opinion of the patient, the general practitioner (GP) and doctor at the ED on the possible preventability of the URP to the ED.

The main aim of this study was to identify the organisational-, technical-, healthcare worker-, patient-, and disease-related root causes involving the acute care chain, that contribute to URPs to the ED within 30 days in patients aged 70 years and older. The secondary aim was to evaluate the potential preventability of these URPs from healthcare worker and patient perspective.

## Methods

### Study design

In this prospective observational study we included 83 older patients (aged ≥70 years) presenting with an URP to the ED of the Amsterdam UMC location VU University Medical Center (VUmc). Patients were included from February 2018 to November 2018. During this period 4133 patients aged 70 years and older visited the ED. In this study we considered an URP as an unplanned return presentation to the ED within 30 days of the initial ED presentation. The Medical Ethics Committee of the VUmc, Amsterdam, approved this study with protocol number 2017.579 on the 13th of November 2017.

### Study setting and population

Patients younger than 70 years old, patients who were unable to give informed consent and critical care room presentations were excluded. We decided to exclude patients living in an assisted living facility or nursing home; they are a different patient population since they already receive continuous institutional care. Patients were included from Monday till Friday during daytime due to the unavailability of researchers outside peak hours; therefore this study consists of a convenience sample. This resulted in a total of 83 included patients returning to the ED within 30 days. There is no valid method to perform a sample size calculation or a power analysis in order to construct a PRISMA profile [13, 14]. However, in the article by Smits et al. [15] they stated that when the number of events is at least 50, the variety of possible unintended events will be captured and a valid causal factor profile can be drawn.

### Data collection

The patients were first approached by the nurses during their ED presentation. The nurse informed whether the patient had visited the ED within the last 30 days and if the patient was potentially eligible for inclusion and interested in participation. Thereafter the potentially eligible patients were approached by the researchers and written informed consent was obtained when they were willing to participate. After informed consent the researcher verified again in the Electronic Patient Record (EPR) if the patients visited the ED in the last 30 days.

Semi-structured interviews were done during the patient’s URP. The interview guide developed for this study is provided as Additional File 1. The aim of this interview was to gather insight into the patient’s home situation, their opinion of the cause of the initial and return ED presentation, the potential preventability of the URP and their motivation for that answer. Furthermore, patients were questioned on medication use, co-morbidity, homecare and hospital admission, ED presentations, GP visits and specialist visits in the year prior to the URP. Regarding their living situation, patients were asked about the types, amount and sufficiency of care they received at home. Additionally, the ‘acute presenting older patient (APOP)’ scores were calculated, based on information concerning age, gender, arrival by ambulance, fall- related visit, ADL support, hospital admission during the last 6 months, and a cognition test. These scores indicate the risk of functional decline and mortality in the next 3 months [16].

Within 3 days of the URP, the attending doctor at the ED and the GP were interviewed by telephone or mail (See Additional File 1). They were asked to give their opinion on whether the ED was the most appropriate location for this patient and the motivation for their answer. Additionally, relevant basic information was collected through the EPR system using a standardized data collection form. The information and the availability of the EPR system is restricted to the hospital. At 6 months follow-up we gathered information for all the patients from the EPR and the GP if necessary, about the frequencies of hospital admission, medical specialist visits and ED return presentations and if the patient was deceased or not. This way we collected a complete dataset.

### Root cause analysis

A root causal tree was developed for the URP using the PRISMA-method (Prevention and Recovery Information System for Monitoring and Analysis) [13]. The PRISMA-method is a root cause analysis method to identify the root causes contributing to an incident or event through the creation of a root causal tree. The PRISMA-method is a structured approach to analyse events as a starting point for improvement and not intended to classify their potential preventability. In this case the return presentation at the ED, was placed at the top of the tree. Direct causes can be identified by constantly asking “why” an event had taken place. These direct causes often have their own causes, the indirect causes. When no further objective causes could be identified, the last indirect cause is considered the root cause. The root causal tree was developed by two independent PRISMA-trained researchers and compared and discussed with a third PRISMA-trained researcher until consensus was reached about the final causal tree. The analysis was based on information from the interviews and the EPR system. The identified root causes were then classified according to the extended version of the Eindhoven Classification Model (ECM) into organisational-, technical-, healthcare worker-, patient-, and disease- related causes [14, 17] (Table 1). PRISMA analysis has previously shown to provide objective and structured insight into causes for (adverse) events and has been accepted by the World Alliance for Patient Safety [13, 14]. The PRISMA-method is not under license.

**Table 1:** Patient characteristics of deceased patients and survival patients

Patient characteristics	Deceased 6 months follow-up ***n*** = 11 (13%)	Survival 6 months follow-up ***n*** = 72 (87%)
**Age**		
**• 70–79, n (%)**	7 (63.6%)	41 (56.9%)
**• 80–89, n (%)**	3 (27.3%)	26 (36.1%)
**• ≥90, n (%)**	1 (9.1%)	5 (6.9%)
***Median (IQR1-IQR3)***	78 (77–84)	78 (73–84)
**Gender Male, n (%)**	9 (81.8%)	39 (54.2%)
**Living with partner, n (%)**	5 (45.5%)	42 (58.3%)
**Homecare, n (%)**	7 (63.6%)	18 (25.0%)
***Median (IQR1-IQR3)***	2 (0–7)	0 (0–0)
**Medication use ≥5, n (%)**	10 (90.9%)	50 (69.4%)
**Admitted to hospital last year, n (%)**	9 (81.8%)	54 (75.0%)
**Hospital admissions**		
**• < year, n (%)**	9 (81.8%)	18 (25.0%)
**• < 6 months, n (%)**	8 (72.7%)	47 (65.3%)
**• < 30 days, n (%)**	8 (72.7%)	37 (51.4%)
**ED presentations last year**		
**• 1, n (%)**	5 (45.5%)	39 (54.2%)
**• 2, n (%)**	3 (27.3%)	16 (22.2%)
**• ≥3, n (%)**	3 (27.3%)	17 (23.6%)
**GP visits last year, median (IQR1-IQR3)**	3 (2–8)	4 (2–5)
**Specialist visit last year, median (IQR1-IQR3)**	6 (2–28)	3 (1–8)
**APOP decline score %** [16]		
**• Low< 60%**	8 (72.7%)	65 (90.3%)
**• High> 60%**	3 (27.3%)	7 (9.7%)
***Median (IQR1-IQR3)***	44 (25–64)	23 (15–39)
**APOP mortality score %** [16]		
**• Low< 60%, n (%)**	11 (100%)	72 (100%)
**•** ***High > 60%,*** **n (%)**	0 (0%)	0 (0%)
***Median (IQR1-IQR3)***	11 (7–44)	6.5 (3–13)
**Days between initial and return ED presentation**		
**• ≤ 2 days, n (%)**	2 (18.2%)	18 (25.0%)
**• 3–7 days, n (%)**	4 (36.4%)	19 (26.4%)
**• > 7 days, n (%)**	5 (45.5%)	35 (48.6%)
***Median (IQR1-IQR3)***	9 (4–19)	7 (2–17)
**Return ED presentation related to initial ED presentation < 30 days, n (%)**	9 (81.8%)	55 (76.4%)
**Triage code, to be seen:**		
**• Directly, n (%)**	0 (0%)	2 (2.8%)
**• Within 10 min, n (%)**	6 (54.6%)	25 (34.7%)
**• > 10 min, n (%)**	2 (18.2%)	28 (38.9%)
**• > 1 h, n (%)**	3 (27.2%)	17 (23.6%)
**Type of referral**		
**• Self, n (%)**	3 (27.2%)	20 (27.7%)
**• GP, n (%)**	4 (36.4%)	30 (41.7%)
**• Specialist, n (%)**	4 (36.4%)	22 (30.6%)
**Arrival by ambulance, n (%)**	7 (63.6%)	15 (20.8%)
**Medical specialty** ^**a**^		
**• Surgery, n (%)**	1 (9.1%)	16 (22.2%)
**• Emergency Medicine, n (%)**	1 (9.1%)	15 (20.8%)
**• Oncology, n (%)**	3 (27.3%)	5 (6.9%)
**• Pulmonology, n (%)**	2 (18.2%)	3 (4.2%)
**• Neurology, n (%)**	2 (18.2%)	7 (9.7%)
**• Internal Medicine, n (%)**	1 (9.1%)	11 (15.3%)
**• Nephrology, n (%)**	1 (9.1%)	1 (1.4%)
**• Cardiology, n (%)**	0 (0%)	2 (2.8%)
**• Gastroenterology, n (%)**	0 (0%)	8 (11.1%)
**• Orthopedics, n (%)**	0 (0%)	1 (1.4%)
**Diagnostics tests at ED** ^**b**^**, median (IQR1-IQR3)**	3 (2–4)	2 (1–3)
**Consultations at ED, median (IQR1-IQR3)**	0 (0–1)	1 (0–1)
**Type of discharge**		
**• Own living environment, n (%)**	3 (27.3%)	35 (48.6%)
**• Admission acute medical unit VUmc, n(%)**	4 (36.3%)	16 (22.2%)
**• Admission other ward VUmc, n (%)**	3 (27.3%)	19 (26.4%)
**• Admission supportive care facility** ^**c**^**, n (%)**	1 (9.1%)	2 (2.8%)

*IQR* Inter Quartile Range, *ED* Emergency Department

^a^ the medical specialty to which the patient is triaged during initial evaluation at the ED

^b^ a single diagnostic test was defined as or a X-ray, or a CT scan, or a MRI, or an ultrasound, or an urine sample or a blood sample used for performing several blood tests

^c^ supporting care facility include rehabilitation centres and elderly care homes

### Potential preventability

Patients, the attending doctors at the ED and the GPs were asked their opinion on the potentially preventability of the URP, and their motivation for that answer. This was asked in the following way: “Do you feel this return ED presentation was potentially preventable in any way by anyone?”. Possible options were “yes”, “no”, “don’t know”, followed by an explanation of their answer.

### Statistics

Data was exported from Castor EDC and imported in IBM SPSS statistics, version 24. Descriptive characteristics and frequencies were calculated, outcomes were presented as frequencies and percentages.

## Results

### Patient characteristics

During the study period, 119 URPs were eligible for inclusion, during the time a researcher was present at the ED. Fourteen patients (12%) were excluded because of planned ED presentations, language barrier, or cognitive impairment. Twenty patients (17%) were eligible for inclusion but refused to participate or were feeling too sick to participate. Patients could only participate once in this study during the study period. Two patients (2%) were already participating in the study and double counted. We therefore only used the data from the first URP for our database. This resulted in a total of 83 included patients. In order to gain insight into the need of care of the deceased patients we divided the patients in two groups: a group of patients that died within 6 months after the URP, and a group of patients that did not die within 6 months of the URP.

In more than half of the total URPs, the patient returned to the ED within 7 days (54% of deceased group and 51% of survival group) and in the majority of patients (82% of deceased group and 76% of survival group) the return presentation was related to the initial presentation. Sixty patients (72%) were referred to the ED, either from a GP (41%) or from a specialist (31%). At 6 months follow-up, the mortality rate was 13% (11 patients), including 4 patients (5%) within the first 30 days. Descriptive statistics of patient characteristics for the deceased and survival group are presented in Table 1.

In the deceased group the majority of the patients (82%) were male, while sexes were represented equally in the survival group (54% male). Deceased patients had a high need and use of care in the last period of their life (Table 1). Most of the deceased patients used 5 or more medications (91% of deceased group and 69% of survival group), the majority received homecare (64% of deceased group and 25% of survival group) and most of them were admitted to the hospital during the last year prior to the return presentation (82% of deceased group and 75% of survival group). The deceased patients presented frequently with an ambulance (64% of deceased group and 21% of survival group) and were triaged with high urgency problems (seen within 10 min 55% of deceased group vs 40% of survival group). During the year preceding the URP the majority of patients had multiple GP visits (median 3 in deceased group and median 4 in survival group) and medical specialist visits (median 6 in deceased group and median 3 in survival group). Additionally, their median APOP decline score was 44% (vs 23% in the survival group) and median mortality score was 11% (vs 7% in the survival group), indicating a 44% risk of functional decline in 3 months and a 11% risk to die within the next 3 months [16].

After 6 months follow-up the deceased group presented one more time to the ED, and when admitted they stayed for 14 days in the hospital, with a range from 0 to 31 days. The survival group presented 0 times to the ED, and when admitted they stayed for 3 days in the hospital with a range from 0 to 70 days. The outpatient clinic was visited 4 times in the survival group and 1 time in the deceased group. Both groups had a median of 1 hospital admission in the follow-up period.

### Root causes

A total of 83 URPs were analyzed and 151 root causes were identified; 19 in the deceased group, 132 in the survival group. The mean number or root causes per URP is 1.8. Twenty-eight URPs (34%) had a single root cause, 43 URPs (52%) had two root causes, 10 URPs (12%) had three root causes and two URPs (2%) had four root causes.

Most root causes were disease-related (*n* = 74, 49%), followed by human-related (*n* = 29, 19%) and organisational- related (*n* = 22, 15%). Table 2 shows the distribution and description of the PRISMA root causes.

**Table 2:** Extended Eindhoven classification model [14, 16, 17]. Distribution of root causes

Main category	Sub category	Code	Description	Deceased patients (*n = 11*) Frequencies (%)	Survival patients (***n*** = 72) Frequencies (%)
**Technical**				0	0
	External	T-ex	Technical failures beyond the control of the organisation.		
	Design	TD	Failures to poor design of equipment etc.		
	Construction	TC	Correct design inappropriately constructed or placed.		
	Materials	TM	Material defects not classified under TD or TC.		
**Organisational**				**2 (10.5%)**	**20 (15.2%)**
	External	O-ex	Failures at an organisational level beyond the control and responsibility of the investigating team.	1 (5.3%)	2 (1.5%)
	Transfer of knowledge	OK	Failure resulting from inadequate measures to train or supervise new or inexperienced staff.		
	Protocols	OP	Failures relating to the quality or availability of appropriate protocols.		2 (1.5%)
	Management priorities	OM	Internal management decisions which reduce focus on patient safety when faced with conflicting priorities.	1 (5.3%)	16 (12.1%)
	Culture	OC	Failure due to attitude and approach of the treating organization.		
**Human**				**3 (15.8%)**	**26 (19.7%)**
	External	H-ex	Human failures beyond the control of the organisation/department		2 (2.5%)
	Knowledge-based behaviour	HKK	Failure of an individual to apply their knowledge to a new clinical situation		
	Qualifications	HRQ	An inappropriately trained individual performing the clinical task		
	Co-ordination	HRC	A lack of task co-ordination within the healthcare team.		7 (5.3%)
	Verification	HRV	Failure to correctly check and assess the situation before performing interventions	1 (5.3%)	6 (4.5%)
	Intervention	HRI	Failure resulting from faulty task planning or performance	1 (5.3%)	11 (8.3%)
	Monitoring	HRM	Failure to monitor the patient’s progress or condition	1 (5.3%)	
**Patient**	Patient-related	PRF	Failures related to patient characteristics or conditions, which are beyond the control of staff and influence clinical progress	**3 (15.8%)**	**16 (12.1%)**
	Disease-related	DRF	Failures related to the natural progress of disease which are beyond control of patient, its carers and staff	9 (47.4%)	65 (49.2%)
**X**	Unclassifiable	X		**2 (10.5%)**	**5 (3.8%)**
**Total**				**19**	**132**

Disease-related factors are related to the natural progression of the disease, implicating that these causes are mostly beyond the control of the patient and the doctor, considering the current circumstances. Examples of root causes are shown in Table 3. Human-related causes originated mostly from intervention (13%), verification (8%) and co-ordination (8%). Management priorities are the most common organisational root causes, representing 11% (*n* = 17) of the total amount of root causes. Nineteen (13%) of the root causes were patient-related. Patient-related factors originated from failures related to patient characteristics or conditions which were beyond the control of hospital staff. The seven unclassifiable root causes (5%) are related to side-effects of medication and closure of the outpatient clinic because of a national holiday. No technical-related root causes were identified in this PRISMA-analysis.

**Table 3:** Examples of root causes

Main category	Subcategory	Example
**Organisational**	External	No check upon the patient by homecare personnel due to shortage of personnel. Due to the late recognition the wound was in a severe state and needed treatment at the ED instead of at the GP.
	Protocol	A switch of antibiotics due to lack of a clear protocol. The patient had no progression of complaints but had to return for the switch.
	Management priorities	Inability to have short-term follow-up diagnostics and to make short-term appointments at the outpatient clinic. Inability to contact the GP.
**Human**	External	An inadequate assessment of the severity of complaints by family of the patient.
	Coordination	The coordination of the location where the patient has to present with his complaint. A well-known patient with anemia, in need of blood transfusions once a week, has to present at the ED instead of going directly to the ward for treatment.
	Verification	The patient contacted the GP by phone, the GP did not visit and assessed the patient at this house but instead advised the patient to go the ED directly.
	Intervention	A delay in diagnose and treatment a patient presenting at the ED with a painful red leg first diagnosed as an infection for which antibiotics were prescribed, returning to the ED because of persisting complaints and pain. During the return presentation it becomes clear that the complaint is not the results of an infection but of a trombo-emboli which needs a different therapy.
	Monitoring	The GP is arranging the transfer for a patient from his house to a rehabilitation center, in the meanwhile the homecare support for this patient is insufficient.
**Patient**	Patient	Refusal of patient to contact the GP before visiting the ED. Refusal of patient to participate with a fall analysis after prior visit, returns at the ED with a second fall.
	Disease	Collapse due to lung embolism, hematuria after operation, respiratory insufficiency by known lung cancer, decompensation cordis after myocardial infarct.
**X**	Unclassifiable	Side-effects of medication, for example chemotherapy. Closure of the outpatient clinic because of a national holiday.

### Preventability

For 39 URPs, a judgement on the potential preventability was available from all three perspectives: the patient, the doctor at the ED and the GP. In total, 14 patients (17%) judged their URP as potentially preventable, the URP was judged as potentially preventable in 21 cases by doctors at the ED (25%) and the GPs judged the URP as potentially preventable in 19 cases (23%). In 18 cases (22%) there was an agreement in the judgement preventable or not preventable between the GP and doctor at ED. In the deceased group they agreed that 1 case was potentially preventable and 1 case was not potentially preventable. In the survival group they agreed that 5 cases were potentially preventable and 11 cases were not potentially preventable. In none of the cases, there was an overall agreement from all three perspectives on the judgement that an URP was potentially preventable.

As shown in Table 4, the perspective from the doctor at the ED was missing in 24 cases (29%) and the perspective from the GP in 18 cases (21%) due to non-response.

**Table 4:** Different perspectives on preventability

Preventability	Yes	No	Don’t know	Missing
**Patient**				
• Deceased	2 (18.2%)	7 (63.6%)	2 (18.2%)	0 (0%)
• Survival	12 (16.7%)	50 (69.4%)	10 (13.9%)	0 (0%)
**Doctor at ED**				
• Deceased	3 (27.3%)	4 (36.3%)	1 (9.1%)	3 (27.3%)
• Survival	18 (25.0%)	28 (38.9%)	5 (6.9%)	21 (29.2%)
**General practitioner**				
• Deceased	2 (18.2%)	3 (27.3%)	1 (9.1%)	5 (45.4%)
• Survival	17 (23.6%)	21 (29.2%)	21 (29.2%)	13 (18.0%)

The reasons patients judged their presentation as potentially preventable were: delayed diagnosis and treatment; persisting complaints after the initial ED presentation when the patient was sent home instead of being admitted to a ward; and refusal or inability of the GP to assess the patient before referral to the ED. In six cases, GPs mentioned that the patient could also (and sometimes even better) be seen at the out-of-hours GP post or visit the outpatient clinic instead of the ED. Additionally, GPs also mentioned the direct contact of the patient with the specialist, thereby bypassing the GP, as a reason for potentially preventable URPs. Doctors at the ED mentioned inability or refusal of the GP to assess the patient before referral; suboptimal or poor communication between healthcare workers; and waiting lists for interventions and operations, as reasons for potentially preventable URPs. Examples of disagreement between patient, GPs and doctors at the ED are provided in Table 5.

**Table 5:** Examples of different perspectives on preventability

Perspectives	Example
Patient VS doctor at ED and GP	A patient, familiar with prostate carcinoma and brain metastases, was recently admitted to the hospital because of neurologic decline due to brain haemorrhage. After discharge, he wakes up in the middle of the night and hears voices. He knows the voices are not real (pseudo hallucinations). He directly presents at the ED. At arrival at the ED the complaint is not there anymore and the doctors relate the complaint to medication side-effects. The doctor at the ED and the GP argued that the patient could have contacted the GP first instead of bypassing him. After assessment of the patient the GP could contact the specialist and they could make a care plan together. When they agree that there would be no alarm symptoms they could decide to not send the patient to the ED. An URP could potentially be prevented. The patient argued that he was scared and connected the complaint with an underlying cause of the brain. Since he was well known in the hospital, it seemed logically for him to present at the ED.
GP VS patient and doctor at ED	A patient presented at the ED after a fall. There were no fractures and the patient was diagnosed with contusions and discharged home. The patient is in pain therefore he receives painkillers and he is limited in mobility. Since the patient received no homecare and lives without partner and has no family to look after him, the advice of the doctor at the ED towards the GP was to arrange supportive care. After 5 days the patients returns to the ED with a fall again. In the 5 days in between there was no additional care arranged. The patient and doctor at the ED argued that the URP was potentially preventable. The GP argued that he frequently recommended home care to the patient over the past year, but the patient refuses to accept additional care. The patient is on a waiting list for a supportive care facility and in the meanwhile he does not allow anyone else entering his home.
Doctor at ED vs patient and GP	A patient presents at the ED with pain in the pelvic region without trauma. He got discharged home with pain medication. Two days later he returns with progression of pain and muscle weakness in the legs and urine incontinency and got admitted with working diagnosis of cauda equine syndrome. The patient and the GP argued that the patient had to be admitted the first time since the pain was not controlled with the medication. The doctor at the ED argued that there was no indication for admission the first time since there were no alarm symptoms back then.

## Discussion

The aim of this study was to identify the root causes that contribute to URPs to the ED within 30 days in patients aged 70 years and older. The secondary aim was to gather more insight in the potential preventability of the URP. The most important finding from our study is that almost half of the root causes (49%) contributing to URPs in older patients are disease-related, followed by human- and organisational- related root causes. Second, in the majority of the URPs (77%), the reason for the return presentation was related to the initial ED presentation and more than half of the patients (52%) returned to the ED within 7 days. In none of the cases there was an overall agreement between the patient and healthcare workers on the judgement that a URP was potentially preventable.

Most studies use the PRISMA-method to focus on the analysis of incidents, unintended events or near-miss events. Similar to Van Galen et al. [18], Cooksley et al. [19] and Fluitman et al. [17], we used the PRISMA-method to gather insight into the root causes of an event, in our study the URP. To our knowledge, the current study is the first study identifying root causes of an URP to the ED with the PRISMA-method. All three other studies concluded that disease-related factors were the majority of the underlying root causes for readmissions. Fluitman et al. [17] conducted a study offering insight into the root causes related to readmissions to the ward and found that healthcare-related root causes were exclusively found in preventable readmissions due to human-related coordination failures and that non-preventable readmission were mostly the result of disease-related factor readmissions. Cooksley et al. [19] analysed readmissions to a tertiary cancer hospital and concluded that all of the readmissions were medical in nature and related to the patient’s malignancy or treatment. PRISMA-analysis demonstrated that four (8%) of the readmissions had active (human) related elements. In the study of van Galen et al. [18] patients were asked to qualify their (probable) reasons for readmission into one or more of the five PRISMA categories: 69% of the root causes were disease-related.

The results of this study are consistent with studies on the nature of ED return presentations where relapse or worsening of medical problems predominate [20] and where the majority or return presentations were related to the initial presentation [20–22]. In addition, the 54% of the patients who returned within 30 days requiring hospital admission is higher than the 3–25% range reported by other studies [3, 23]. Our findings show that the percentage of URPs judged as potentially preventable was low and there was no consensus among the patient and healthcare workers about the preventability. This is similar to the results of Suffoletto et al. [12] who concluded that concordances between patients, their caregivers and emergency physicians on the preventability of the ED visit within 30 days of hospital discharge were poor and there were significant discordances in identifying an intervention in order to potentially prevent the ED visit. In line with de Gelder et al. [24], our results show that patients returning to the ED are in a high need of care. This could be a sign that there is more going on than their medical health issues alone. De Gelder et al. concluded that an early ED revisit can be considered as a predictor of functional decline or mortality, based on geriatric parameters (for example polypharmacy) and cognition tests. Male sex, polypharmacy and cognitive impairment were associated with and a predictor of an 30 day revisit [24]. Similarly to our results, the patients in the deceased group were mostly male and had high APOP score and medication use.

There is a lack of patient information and awareness regarding the appropriate type of care in the appropriate location. The current Dutch healthcare system due to government restrictions, does not have a suitable alternative to meet the perceived needs of some patients, for example due to the strict allocation of homecare to patients and the reduction of nursing home beds. Our study shows that half of the root causes are disease–related and the majority of the URPs was judged as not preventable, this may indicate that there are not many options to prevent the URP. However, the question remains if the root cause which is classified as disease-related is due to suboptimal care and treatment during the initial ED presentation and the follow-up care or to worsening of a known chronic disease. An URP caused by a disease-related factor should trigger healthcare workers to look critically into the process of care and focus on the older patients’ specific and multifaceted needs, their experience and relevant outcomes. The process of care should be an individual process and depends on the circumstances and the stage of life the patient is in. Healthcare workers should discuss in an early stage if advanced care planning (ACP) is indicated, in order to give the patient the optimal appropriate care and potentially reduce the number of URPs. In our study we didn’t specifically ask patients or their doctors about goals of care and whether they had an advanced care plan. It is important to realise that in our study not all patient with an URP had an advanced care plan.

ACP involves a process identifying personal preferences concerning goals of care and translating those values into medical care plans. ACP is associated with increased satisfaction in end-of-life care as well as reducing inpatient hospitalisation, reducing in-hospital deaths and increasing hospice use [8, 23]. In order to make ACP successful, it is important that patients, caregivers and family are involved. An example of ACP is the case with an older patient with an advanced neurologic disease, having a high risk of function decline and mortality, who presents frequently at the ED, with a complaint due to progression of disease. Questions about the treatment plan and care regarding quality of life and personal values arise. After consultation with the patient, his family and his caregivers it can be decided to change the treatment plan and to not present at the ED with a complaint but to stay at home and control disease-symptoms in the primary care with help of the GP and home care. Additionally, there is literature that highlights the extensive array of health care services delivered at the end of life and the high costs associated with care. For example in the United States, about one quarter of all Medicare spending goes toward care for people during their last year of life [8]. Timing ACP is urgently needed to control and reduce costs and address ineffective and irrational healthcare utilization, especially at the ED. In addition, improved communication in the chain and a clear and safe inter-professional discharge plan may also lead to less URPs. Perhaps substantial reorganization of the acute care chain could help to reduce the URPs, for example with the addition of more emergency outpatient clinics. It would be interesting for future research to focus on earlier detection of the disease-related root causes to determine their contribution to the potential preventability of an URP. Furthermore it would also be interesting to focus on the role of an early and appropriate use of ACP in these cases.

The strength of this study includes the prospective design, the semi-structured interviews, the follow-up period and the involvement of the complete acute care chain. This study also has some limitations. First, the causal trees of some patients are possibly incomplete due to unresponsiveness of the healthcare workers. This may have resulted in an underestimation of the number of root causes and limited the opportunity to draw conclusions when combining the opinions of all stakeholders. Second, our sample is not a representative sample compared to patients visiting the ED 24–7, since we only included patients when they were present at the ED on Monday till Friday during daytimes and excluded patients who were not living independently. However, we have no reason to postulate that patients who presented outside these time limits were any different to the included patients. Third, we are aware of the relatively small cohort of patients. Therefore it was not possible to perform reliable statistical analyses to test for significance between subgroups. The aim of our study, however, was to explore root causes of URPs and judgements on potential preventability. Fourth, in most cases, there was a relation and therefore dependency between the initial ED presentation and the URP. Therefore, it may be questioned whether the root causal profile of the URPs is independent of the root causal profile of initial ED presentations. Although this study focused on the contributing causes for URPs, there may be interactions with the initial ED presentations. Furthermore we are aware of the bias due to the participants role; a patient who is a self-referral is very likely to judge the URP as not preventable.

## Conclusion

Half of the patients who returned to the ED, returned within the first 7 days. In most cases the URP was related to the initial presentation. Disease-related factors are the most common root cause for the URP and our results indicate that an URP should trigger healthcare workers to look further into the patient’s process of care and focus on their specific and multifaceted needs. The majority of the URPs was judged as not preventable. However, it would be interesting for future research to focus on earlier detection of the disease-related root causes to determine their contribution to the potential preventability of an URP.

## Supplementary information

**Additional file 1.** Interview guide

## Data Availability

The datasets used and/or analysed during the current study are available from the corresponding author on reasonable request.

## Abbreviations

ACP: Advanced care planning
APOP: Acute presenting older patient
ECM: Eindhoven classification model
ED: Emergency department
EPR: Electronic patient record
GP: General practitioner
IQR: Inter quartile range
PRISMA: Prevention and recovery information system for monitoring and analysis
URP: Unplanned return presentation
