# Supplementary material for: Unplanned return presentations of older patients to the emergency department: a root cause analysis
Source: BMC Geriatr. 2020 Sep 22;20:365. doi: 10.1186/s12877-020-01770-x (PMC7510142; doi:10.1186/s12877-020-01770-x)
Supplement: Supplementary file 1 — Additional file 1. Interview guide [file 12877_2020_1770_MOESM1_ESM.pdf]

Interview guide-patient

Number participant	…………….
Age	……………. years
Gender	0 = man
	1 = woman
Shared household	0 = no (alone)
	1 = yes (married or living together)
Living situation	0 = independent
	1 = sheltered housing
	2 = supportive care facility
	3 = nursing home
	4 = revalidation home
Help with household activities	0 = no
	1 = family member or caregiver
	2 = domestic help
Frequency help with household activities	……………. times per week
Home nursing	0 = no
	1 = yes
Frequency of home nursing	……………. times per week

Number of medications patient uses	…………….
Number of comorbidities patient	…………….
Frequency of hospital admissions year prior to this ED presentations	……………. times
Frequency of ED presentations year prior to this ED presentations	……………. times
Frequency of GP visits yea prior to this ED presentations	……………. times
Frequency of specialist visits year prior to this ED presentations	……………. times

Opinion on sufficiency of provided care	0 = inadequate<br>1 = adequate<br>2 = more than adequate<br>3 = good
How often did you fall last year?	……………. times
Was this ED presentation potentially preventable?	0 = no<br>1 = yes<br>2 = I don’t know

Why was it, why wasn’t it?

- Are you completely recovered after the initial ED presentation?
- Did something changed in your care situation since the initial ED presentation?
- Were you fully committed to the proposed therapy after the initial ED presentation?
- Was the aftercare well arranged after the initial ED presentation and are the involved healthcare professionals informed?
- Did you visit the GP before the ED with your complaint?
- Who is responsible for what part of your care plan? (patient, partner, caregiver, home care facility)
- Do you know with which problems you can present at the GP and with which problems you can present at the hospital?
- In general do you have a good therapy compliance?

Interview guide-doctor at ED

What is in your opinion the reason of this return presentation at the ED of this patient?	………………………………………………………………………………<br>………………………………………………………………………………<br>………………………………………………………………………………<br>………………………………………………………………………………
Is the ED the most appropriate location for this patient?	0 = no<br>1 = yes
Is this ED return presentation appropriate?	0 = no<br>1 = yes
Could the patient have been treated at a different location than the ED?	………………………………………………………………………………<br>………………………………………………………………………………<br>………………………………………………………………………………<br>………………………………………………………………………………
Was this ED return presentation potential preventable?	0 = no answer<br>1 = I don't know<br>2 = no<br>3 = yes
In which way could this ED return presentation potentially be prevented?	………………………………………………………………………………<br>………………………………………………………………………………<br>………………………………………………………………………………<br>………………………………………………………………………………

Interview guide-GP

Is the ED the most appropriate location for this patient?	0 = no<br>1 = yes
Is this ED return presentation appropriate?	0 = no<br>1 = yes
Was this ED return presentation potential preventable?	0 = no answer<br>1 = I don't know<br>2 = no<br>3 = yes
In which way could this ED return presentation potentially be prevented?	………………………………………………………………………………<br>………………………………………………………………………………<br>………………………………………………………………………………<br>………………………………………………………………………………
Could the patient have been treated at a different location than the ED?	………………………………………………………………………………<br>………………………………………………………………………………<br>………………………………………………………………………………<br>………………………………………………………………………………
Was there an adequate discharge plan after the initial ED presentation?	0= no<br>1= yes
Was there an follow-up appointment at the hospital or at the GP after the initial ED presentation?	0 = no<br>1 = yes, explanation:<br>………………………………………………………………………………<br>………………………………………………………………………………<br>………………………………………………………………………………<br>………………………………………………………………………………
Were you aware of the patients health status after the initial ED presentation?	0 = no<br>1 = yes
Use of preventive diagnostics	0 = no<br>1 = yes
Type of preventive diagnostics	0 = blood tests<br>1 = control appointment<br>2 = other:……………………………………………………………

Frequency of preventive diagnostics	……………. times a year
Does the patient have comorbidities?	0 = no<br>1 = yes; number of comorbidities: …………….
Number of medications patient uses	…………….
Is the patient monitored by the GP,<br>if yes on what way?	0 = no<br>1= yes, explanation:<br>………………………………………………………………………………<br>………………………………………………………………………………<br>………………………………………………………………………………<br>……………………………………………………………………………….
How is the patients mobility?	………………………………………………………………………………<br>………………………………………………………………………………<br>………………………………………………………………………………<br>……………………………………………………………………………….
Is there an increased risk of falling?	0 = no<br>1 = yes
Does the patient have cognitive impairments?	0 = no<br>1 = yes
What is the patients living situation, is it stable, is the patient receiving home care of help with house hold activities?	………………………………………………………………………………<br>………………………………………………………………………………<br>………………………………………………………………………………<br>………………………………………………………………………………
